# Back pain in adolescent idiopathic scoliosis: frequency and risk factors

**DOI:** 10.1007/s43390-024-00904-0

**Published:** 2024-06-23

**Authors:** Stefan Sarkovich, Claudia Leonardi, Matthew Darlow, Davis Martin, Peter Issa, Tara Soria, Amy Bronstone, Carter Clement

**Affiliations:** 1 Department of Orthopaedics, Louisiana State University Health Sciences Center, New Orleans, LA USA; 2 School of Medicine, Louisiana State University Health Sciences Center, New Orleans, LA USA; 3 Behavioral & Community Health, LSU Health Sciences Center, New Orleans, LA USA; 4 Graduate Medical Education, HCA Florida Bayonet Point Hospital, 14000 Fivay Rd, Hudson, FL 34667 USA

**Keywords:** Adolescent Idiopathic scoliosis, Back pain, AIS

## Abstract

**Purpose:**

Although back pain is commonly reported in patients with adolescent idiopathic scoliosis (AIS), factors that influence the presence and severity of back pain in AIS, including curve-specific variables, have not been well studied. This study aims to describe the prevalence and severity of back pain in AIS patients and determine the extent to which patient characteristics, including curve-specific factors, are associated with a higher risk of back pain in AIS.

**Methods:**

The study was a retrospective medical records review of adolescents (aged 10–17 years) diagnosed with AIS between 01/01/2018 and 12/31/2021 at an academic tertiary children’s hospital. Patients with previous spine surgery were excluded. Variables collected included demographics (age, sex, race, insurance), Lenke classification, major coronal curve, back pain-related information, Risser stage, vitamin D levels, post-diagnosis brace utilization, physical therapy or chiropractic treatment, and surgery.

**Results:**

A total of 891 AIS patients were included in the analysis. The sample was predominantly female (73.3%) and insured by Medicaid (57.8) with a mean age of 12.8 years. The mean major coronal curve was 26.3 degrees. Most patients had Lenke type 1 (47%) and type 5 (41%) curves. Nearly half of patients reported back pain (48.5%) with average pain severity in the low-to-moderate range (4.7) on FACES pain scale (0–10). Among those who reported back pain, 63.2% specified a location with the majority reporting pain in the lumbar region (56%) and, less commonly, in the thoracic (39%) and scapular (8%) regions. Lumbar pain was associated with significantly higher pain intensity (p = 0.033). Additionally, the location of pain reported was associated with location of major coronal curve (p < 0.0001). No association was observed between pain presence and vitamin D deficiency (p = 0.571, n = 175), major coronal curve magnitude (p = 0.999), Lenke curve type (p = 0.577), and sex (p = 0.069). Older patients, those insured by Medicaid, and those with higher Risser scores were more likely to report pain scores (p = 0.001 for all).

**Conclusion:**

Nearly half (48%) of newly diagnosed AIS patients experience back pain which is higher than the prevalence of 33% seen in the general adolescent population. Pain was more prevalent among patients over the age of 13, with heavier body weight, and those insured by Medicaid. Pain was most commonly reported in the lumbar region, especially among patients with lumbar curves. This information can be helpful in counseling AIS patients, though further investigations are needed, especially to determine the underlying causes of back pain in AIS and to elucidate the discrepancy in pain between patients with Medicaid and commercial insurance.

**Level of evidence.:**

Prognostic Study Level II.

## Introduction

Adolescent idiopathic scoliosis (AIS) is a common condition affecting the adolescent population with an overall prevalence ranging from 0.5% to 5.2% [1]. Back pain is common in adolescents with AIS, with a prevalence ranging from 23 to 68% [2, 3], which is more than two-fold higher than in adolescents without AIS [2–5]. Because back pain in AIS can negatively affect function/activity, mental health, self-image, quality of life in adolescents [6–8], can contribute to absenteeism from school and work in adolescents and young adults [9], and often becomes chronic into adulthood [10], it is critical to identify and understand potential risk factors associated with back pain presence and severity.

Although various biomechanical, biochemical, and neuropsychological factors have been identified as contributing to back pain in AIS, its root cause is unclear in many cases [10]. Back pain in AIS is associated with a discrete underlying pathology in only 9–35% of AIS patients [4, 11, 12], and there is no correlation between back pain intensity and the presence of an underlying pathology identified on MRI [11]. There is a paucity of evidence regarding the extent to which curve-specific factors influence back pain in nonoperative AIS cases. One study analyzed predictors of back pain in AIS patient who were surgical candidates and found a positive correlation between severity of back pain and lumbar curve rigidity [13]. All other studies examining the relationship between curve type and back pain were conducted in operative AIS patients and produced inconsistent findings [14–16]. Thus, there is a lack of consensus on whether curve magnitude or location influences preoperative and postoperative pain scores in patients with AIS [10].

We conducted a retrospective chart review to describe the prevalence and nature of back pain and examine the relationship between curve-specific factors and back pain in newly-diagnosed AIS patients. The study was conducted at a setting with a racially and socioeconomically diverse patient population in the United States (US).

## Materials and methods

An IRB approved retrospective chart review was conducted examining patients newly diagnosed with AIS at an academic tertiary free-standing children’s hospital from January 1, 2018 to December 31, 2021. Patients aged 10–17 years who had been diagnosed with AIS by a board-certified pediatric orthopedic surgeon were included in the study; patients who had been diagnosed with AIS at other institutions were excluded. Additional exclusion criteria were previous spine surgery or spinal trauma.

Datapoints collected included demographics (age, sex, race, insurance), clinical characteristics at AIS diagnosis (weight, Lenke classification curve type, family history of scoliosis, vitamin D level, Risser score), and treatment (physical therapy or chiropractic treatment (PT/CT), bracing, or surgery) received during the follow-up period which ranged from 9 to 57 months. If back pain was mentioned in the chart or there was documentation of a score ≥ 1 on the Wong-Baker FACES Pain Rating Scale (FACES), the patient was considered to have back pain. The FACES scale shows a series of faces ranging from a happy face at 0 (“no hurt”) to a crying face at 10, which represents “hurts like the worst pain imaginable” [17]. The FACES scale was administered by a nurse or nurse assistant in a face-to-face interview at the initial clinic visit and has demonstrated adequate reliability and validity for the assessment of pain intensity in children and young adults [18]. The location of back pain (lumbar, thoracic, scapular) was captured if reported in the chart.

Data was collected and managed using REDCap and analyzed using SAS version 9.4 (SAS Institute Inc, Cary, NC, USA). Univariable associations between pain and categorical variables were investigated using the chi-square test and between pain and continuous normally-distributed variables using the Student’s t-test. Multivariable mixed logistic regression was used to examine the association between pain and characteristics shown to be associated with pain (p < 0.10) in univariable analyses. The fixed effects included in the model were age, sex, weight, Risser scale, insurance, and treatment. Year of diagnosis was included as a random effect to account for possible variability over time. A p-value of less than 0.05 was considered statistically significant.

## Results

A total of 891 patients newly diagnosed with AIS were included in the study. Patient demographic and clinical characteristics are shown in Table 1. The mean age at AIS diagnosed was 12.8 years. Most patients were female (73.3%) and insured by Medicaid (57.8%). Race was self-reported as White or Caucasian by 47.3%, Black or African-American by 37.9%, and “other” by 8.6% of patients. The average major coronal curve at diagnosis was 26.3° (range, 10–90°) with most curves classified as Lenke class 1 (45.4%) and 5 (44.1%).

**Table 1:** Patient demographics and clinical characteristics (n = 891)*

Characteristic	
Age, years (mean, SD)	12.8 (1.5)
Sex, % (n)	
Female	73.3 (653)
Male	26.7 (238)
Race, % (n)	
Black or African American	37.9 (338)
White or caucasian	47.3 (421)
Other	8.6 (77)
Declined to report	62 (55)
Insurance, % (n)	
Medicaid	57.8 (515)
Private	39.5 (352)
Self-pay	2.7 (24)
Weight, kg (mean, SD)	59.6 (18.1)
Vitamin D, ng/ml (mean, SD)	26.3 (12.2)
Family history of scoliosis, % (n)	17.2 (151)
Cobb angle at AIS diagnosis,° (mean, SD)	26.3 (15.5)
Scoliosis curve location, % (n)	
Lumbar	9.1 (81)
Thoracic	31.1 (277)
Thoraco-Lumbar	28.3 (252)
Combined	31.5 (281)
Lenke classification—curve type, % (n)	
1	45.4 (404)
2	3.5 (31)
3	1.0 (9)
4	0.8 (7)
5	44.1 (392)
6	5.2 (46)
Risser stage, % (n)	
0–3	64.2 (569)
4 +	35.9 (318)

^*^Data were missing for weight (n = 14), family history of scoliosis (n = 14), vitamin D level (n = 716), Risser stage (n = 4), and Lenke classification (n = 2)

*AIS* adolescent idiopathic scoliosis, *SD* standard deviation

**Fig. 1:**
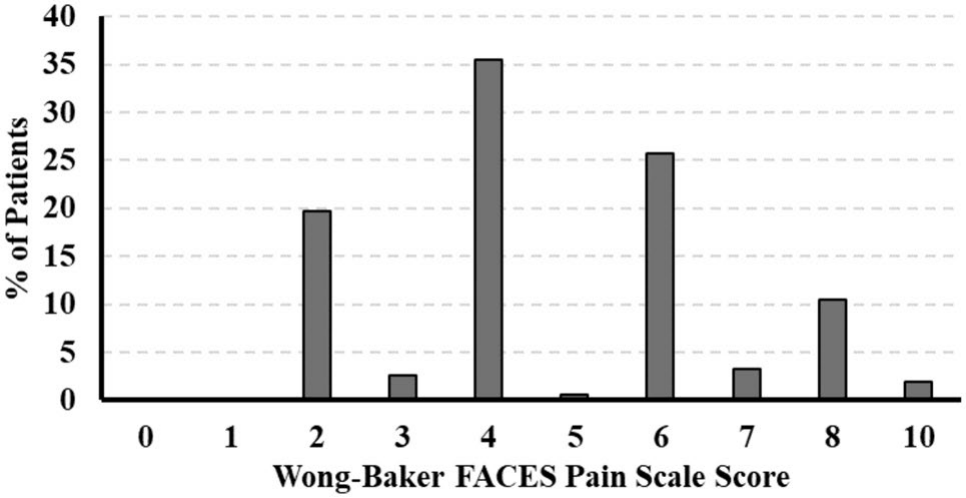
Distribution of scores on the wong-baker FACES scale (Range, 0–10) among patients who reported back pain (n = 152)

Almost half of the patients reported back pain (48.5%, 432/891). Of the 432 patients who reported back pain, the FACES scale was administered to 152 patients (35%) who had an average score of 4.7 (range, 2–10) indicating low-to-moderate pain intensity on average (Fig. 1). There were no statistically significant associations between administration of the FACES scale and patient characteristics (Table 2).

**Table 2:** Associations between wong-baker FACES pain scale administration and patient characteristics (n = 432)

Characteristic	FACES Not Administered (n = 280)	FACES Administered (n = 152)	p-value
Age, years (mean, SEM)	13.1 (0.1)	13.1 (0.1)	0.989
Sex, % (n)			0.955
Female	76.1 (213)	76.3 (116)	
Male	23.9 (67)	23.7 (36)	
Race, % (n)			0.258
Black or African American	39.3 (110)	46.1 (70)	
White or caucasian	46.8 (131)	43.4 (66)	
Other	8.5 (24)	8.5 (13)	
Declined to report	5.4 (15)	2.0 (3)	
Insurance, % (n)			0.670
Medicaid	64.7 (181)	63.2 (96)	
Private	32.1 (90)	34.9 (53)	
Self-pay	3.2 (9)	2.0 (3)	
Weight, kg (mean, SEM)	62.5 (1.1)	59.9 (1.5)	0.167
Cobb angle at AIS diagnosis, ° (mean, SEM)	27.1 (0.9)	25.1 (1.2)	0.192
Risser stage, % (n)			0.396
0–3	58.8 (163)	54.6 (246)	
4 +	41.2 (114)	45.4 (69)	

*AIS* adolescent idiopathic scoliosis, *SEM* standard error of the mean

**Fig. 2:**
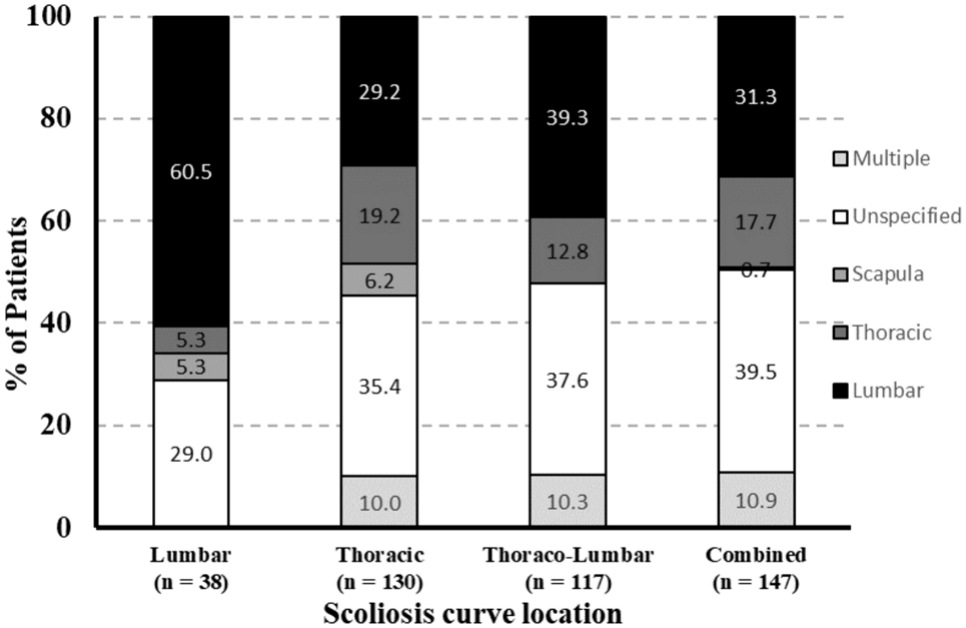
Pain location associated with scoliosis curve location among patients who reported back pain (n = 432)

The location of back pain was indicated in the chart for 273 of 432 (63.2%) patients with documented pain. Among the patients who reported one or more pain locations, the most common location of pain was the lumbar region only (56.0%; 153/273) followed by the thoracic region only (24.9%; 68/273), a combination of locations (15.0%, 41/273; lumbar and thoracic, n = 30; thoracic and scapula, n = 6; lumbar and scapula, n = 2, lumbar, thoracic and scapula, n = 3) and lastly scapular region only (4.0%; 11/273). In a multivariable model including each pain location as a binary explanatory variable and pain score as outcome (n = 152), pain in the lumbar region was associated with a higher average pain intensity score than no pain in the lumbar region (4.9 vs. 4.2, p = 0.033). Individuals with thoracic pain or scapular pain did not have higher mean FACES scores than patients without pain in these regions (thoracic: 4.3 vs. 4.8; p = 0.234; scapular: (4.5 v. 4.6, p = 0.835). Furthermore, the location of pain was associated with the location of the major spinal curve (p < 0.0001, Fig. 2). Among patients diagnosed with a lumbar scoliosis curve, the majority (60.5%, 23/38) reported pain primarily in the lumbar region. In contrast, patients with other curve locations exhibited a higher percentage of unspecified pain location, pain in multiple locations, and thoracic pain (Fig. 2).

Among patients with back pain (n = 432), pain was documented for 69.2% (299/432) at initial AIS diagnosis, 9.0% (39/432) within 6 months of AIS diagnosis, 7.4% (32/432) between 6 and 12 months of AIS diagnosis, and for 14.4% (62/432) more than 1 year after diagnosis.

Table 3 shows the univariable associations between the presence of back pain and patient characteristics. Race was associated with back pain when all patients were included (p = 0.026); however, when patients who declined to report race were excluded, the association was no longer statistically significant (p = 0.202). Back pain was associated with insurance (p = 0.001) such that patients who reported back pain were more likely to be insured by Medicaid (64.1% vs. 51.9%) and less likely to carry private insurance (33.1% vs. 45.5%) compared to patients not reporting pain. Patients with back pain were significantly older (13.1 years vs. 12.6 years, p < 0.0001), had a higher mean body weight (64.1 k vs. 57.8 kg, p = 0.002) than those without back pain and a higher percentage of patients with a Risser score of 4 or more (42.7% vs. 29.5%, p < 0.001). Sex (p = 0.060), Cobb angle (p = 0.999), vitamin D level (p = 0.571), major curve location (p = 0.489), Lenke classification (p = 0.570), and family history of scoliosis (p = 0.269) were not significantly associated with the presence back pain. No correlation was observed between pain severity (FACES score) and the Cobb angle (ρ = 0.044, p = 0.593, n = 152).

**Table 3:** Univariable associations between self-reported back pain and patient characteristics (n = 891)*

Characteristic	No Back Pain Reported (n = 459)	Back Pain Reported (n = 432)	p-value
Age, years (mean, SEM)	12.6 (0.1)	13.1 (0.1)	< 0.0001
Sex, % (n)			0.060
Female	70.6 (324)	76.2 (329)	
Male	29.4 (135)	23.8 (103)	
Race, % (n)			0.029
Black or African American	34.4 (158)	41.7 (180)	
White or Caucasian	48.8 (224)	45.6 (197)	
Other	8.7 (40)	8.6 (37)	
Declined to report	8.1 (37)	4.2 (18)	
Insurance, % (n)			0.001
Medicaid	51.9 (238)	64.1 (277)	
Private	45.5 (209)	33.1 (143)	
Self-pay	2.6 (12)	2.8 (12)	
Weight, kg (mean, SEM)	57.8 (0.9)	61.6 (0.9)	0.002
Vitamin D, ng/ml (mean, SEM)	25.7 (1.4)	26.8 (1.2)	0.571
Family history of scoliosis, % (n)	18.6 (84)	15.8 (67)	0.269
Cobb angle at AIS diagnosis, ° (mean, SEM)	26.3 (0.7)	26.4 (0.7)	0.999
Major curve location, % (n)			0.489
Lumbar	9.4 (43)	8.8 (38)	
Thoracic	32.0 (147)	30.1 (130)	
Thoraco-Lumbar	29.4 (135)	27.1 (117)	
Combined	29.2 (134)	34.0 (147)	
Lenke classification—Curve type, % (n)			0.570
1	43.6 (200)	47.4 (204)	
2	2.8 (13)	4.2 (18)	
3	0.9 (4)	1.2 (5)	
4	0.7 (3)	0.9 (4)	
5	46.6 (214)	41.4 (178)	
6	5.5 (25)	4.9 (21)	
Risser stage, % (n)			< 0.0001
0–3	70.5 (323)	57.3 (246)	
4 +	29.5 (135)	42.7 (183)	

^*^Data were missing for weight (n = 14), family history of scoliosis (n = 14), vitamin D level (n = 716), Risser stage (n = 4), and Lenke classification (n = 2)

**Fig. 3:**
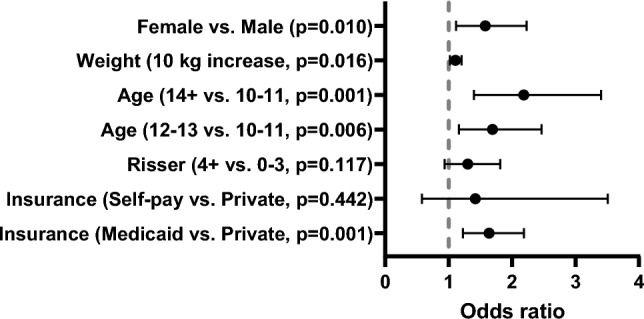
Patient characteristics associated with back pain in AIS. The figure is a forest plot displaying the odds of back pain derived from a multivariable logistic regression (n = 830)

Figure 3 shows the results of a multivariable logistic regression for presence of back pain. Older patients were significantly more likely to have back pain: patients aged 12–13 years were 1.7 times more likely to have back pain than those aged 10–11 years (adjusted odds ratios [aOR] = 1.65; 95% confidence interval [CI] 1.12–2.44; p = 0.006) and patients aged ≥ 14 were 2 times more likely to have back pain than those aged 10–11 years (aOR = 2.2; 95% CI 1.18–3.00; p = 0.001). Females and patients with Medicaid were 1.6 times more likely to have back pain than males and patients with private insurance, respectively (sex: aOR = 1.58; 95% CI 1.18- 3.00; p = 0.010; insurance: aOR = 1.64; 95% CI 1.14–2.09; p = 0.001).

**Fig. 4:**
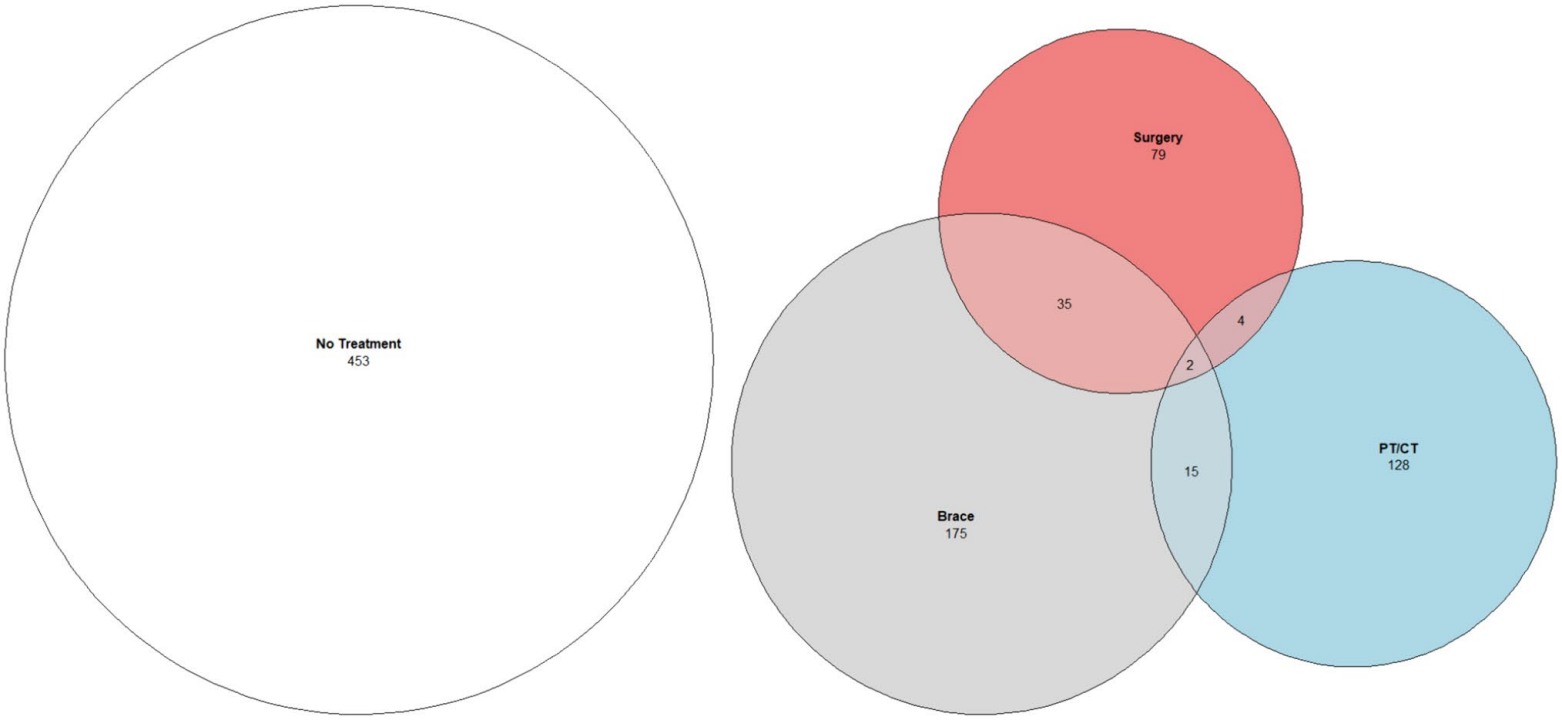
Venn diagram of treatments received after adolescent idiopathic scoliosis diagnosis (n = 891). *CT* chiropractic treatment, *PT* physical therapy

**Fig. 5:**
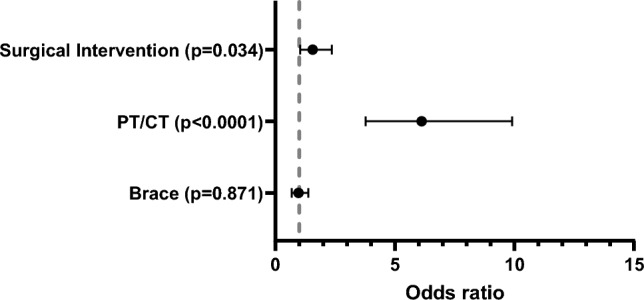
Association between treatment received after AIS diagnosis and pain. Forest plot displays the odds ratio of reporting pain (FACES score > 0) based on a multivariable logistic regression analysis in patients diagnosed with AIS (n = 830). *AIS* adolescent idiopathic scoliosis. *PT/CT* Physical Therapy or Chiropractic Treatment

Nearly half of patients (49.1%, 438/891) received PT/CT, bracing, and/or surgery following AIS diagnosis. In the overall sample (n = 891), 14% (125/891) of patients were treated with bracing only, 12% (107/891) with PT/CT only, 4.3% (38/891) with surgery only, and 6.3% (56/891) with combinations of these treatments (bracing and PT/CT, n = 15; PT/CT and surgery, n = 35; PT/CT plus surgery, n = 4; bracing, PT/CT and surgery, n = 2)(Fig. 4). As shown in Fig. 5, pain was associated with receiving PT/CT and surgery after AIS diagnosis: patients who received PT/CT were 6.4 times more likely to report back pain than those who did not receive PT/CT (aOR = 6.38; 95% CI 3.72–11.0; p < 0.0001) and patients who underwent surgical intervention were 1.6 times more likely to report back pain than those who did not undergo surgery (aOR = l.59, 95% CI 1.12–2.40, p = 0.034). Prescription of a thoracic lumbar sacral orthosis brace was not significantly associated with the presence of back pain. In a multivariable model, patients who received PT/CT reported higher average FACES scores than those who did not receive PT/CT (5.5 vs. 4.8, p = 0.043). Surgery and bracing were not significantly associated with the FACES score (p = 0.400 and p = 0.269, respectively).

## Discussion

In the present study, nearly half (48.5%) of patients with AIS treated at an academic tertiary children’s hospital reported back pain, with lumbar pain being the most common location of reported pain. Additionally, lumbar pain was significantly associated with higher pain intensity compared to thoracic or scapular pain. Several curve-specific and patient-specific characteristics were associated with back pain, including Medicaid insurance, older age, patients with higher body weight, and more advanced skeletal maturity as determined by Risser stage. Patient with lumbar scoliosis were more likely to report lumbar pain, and patients with thoracic scoliosis were more likely to report thoracic pain. Curve magnitude, Lenke Type, sex, and vitamin D deficiency were not associated with back pain. Unsurprisingly, patient reporting pain were more likely to be referred to PT/CT, but pain was not associated with likelihood of bracing or surgery on multivariable analysis.

Although AIS alters the 3-dimensional configuration and biomechanics of the spine, the relationship between curve types and curve magnitude (ie. major coronal curve) with back pain is not well understood. Several studies have demonstrated that the magnitude of the curve does not correlate with experiencing back pain [4, 19, 20], while others demonstrate a positive association between curve magnitude and pain [21, 22]. We found no association between back pain and Lenke curve type or the magnitude of the major coronal curve. However, there was a significant association with the location of pain reported and the location of the major coronal curve. Specifically, curves in the thoracolumbar and lumbar regions had a higher prevalence of AIS back pain than thoracic curves [23]. These conflicting results suggest that the origins of back pain may be due to abnormalities other than the curve magnitude such as decreased trunk strength or lack of flexibility in the hip flexors and hamstrings. We suspect muscle dysfunction and higher BMI causing increased asymmetrical pressure on facet joints to be the principal factors causing pain rather than curve magnitude.

Consistent with other studies [2, 24], most back pain in this study occurred in the lumbar and thoracic regions and was moderate in severity on average [5, 24, 25]. Patients with lumbar pain reported more severe pain than patients with pain in other regions. The lumbar region of the spine plays a significant role in weight-bearing, moving, twisting, and bending. When 3-dimensional configurational changes occur in the spine such as in AIS, these forces can be magnified and may therefore contribute to AIS patients experiencing lumbar back pain more often.

Consistent with previous research [4, 8, 13, 26, 27], we found that older age was associated with a higher prevalence of back pain in adolescents with AIS. The association of older age and back pain may be related to heavier average patient weight, causing higher degrees of asymmetric loading, resulting in higher rates of reported back pain. The present study also showed a disparity in self-reported back pain among AIS patients with Medicaid versus private insurance. Patients with Medicaid were 1.6 times more likely to have back pain than patients with private insurance. Medicaid enrollees are more likely to be socioeconomically disadvantaged compared with patients with private insurance and to have less access to spine care [28]. Improving access to care for socioeconomically disadvantaged patients is an important goal that may reduce disparities in back pain among AIS patients.

The majority of patients newly diagnosed with AIS in the present study were managed conservatively. Pain appeared to be an important driver of physical therapy but not bracing or surgery. There is a paucity of data regarding the effectiveness of conservative AIS treatments such as bracing and physical therapy on pain. Randomized clinical trials suggest that bracing does not improve quality of life during treatment or over time [29]. The use of bracing as the sole conservative treatment was associated with lower disability scores in one study [30], but with significant limitations in everyday activities and neck-related impairment in another study [3]. A randomized clinical trial showed that physical therapy consisting of Schroth scoliosis-specific exercises over 6 months improved pain, self-image and back muscle endurance in patients with AIS with curves 10–45° [31], and a retrospective chart review found that adolescents with AIS and Cobb angles of 20–40° who completed Schroth scoliosis-specific exercises had improved health-related quality of life and radiographic parameters after treatment [32]. Although additional studies are needed to corroborate these findings, clinicians should consider referring AIS patients with back pain and curves < 45° to physical therapy.

In contrast to a previous study that reported a correlation between vitamin D deficiency and back pain in adults [33], the present study found no significant relationship between these variables in our AIS population. Similarly, Beling and colleagues demonstrated that vitamin D levels do not influence AIS patients’ experience of pain before or after surgical intervention [34]. However, a contrasting study by Hampton et al. examining patients with AIS demonstrated a strong correlation between preoperative back pain scores and vitamin D deficiency [35]. The present study is the first to our knowledge to examine the relationship between Vitamin D deficiency and pain in a population including both operative and nonoperative patients with AIS. Additionally, although family history suggests a genetic connection with AIS [36], our results demonstrate that family history of scoliosis was not associated with increased pain.

This study has several limitations. First, data were obtained from a single pediatric hospital and, thus, may not be representative of other hospital systems. Second, the retrospective design precludes determinations of causality. Third, medical record data were missing for some patients. Only 152 of 891 patients in this study had Wong-Baker FACES pain scales completed during evaluation. Although comparisons of characteristics between patients with and without FACES scores did not reveal any statistically significant differences (Table 2), it is possible that these groups systematically differed in some way. Our institution has implemented operational changes to improve the consistency of patient-reported outcomes data collection. Currently, all patients who present to the clinic for AIS initial evaluation or follow-up are administered the FACES and the Scoliosis Research Society-22 questionnaire by a nurse or medical assistant prior to seeing the physician; these data are then uploaded to a secure electronic medical record. Although this study included only a single measure of pain, the FACES score is well correlated with other measures of pain severity validated for use in pediatric populations, such as the visual analog scale and numerical rating scale [37]. Lastly, some variables of interest were not captured in the medical records, such as pain frequency, pain aggravating/alleviating factors, the effectiveness of PT for treating pain, pain interference in daily activities, and mental health diagnoses (e.g., depression, anxiety).

## Conclusion

We examined a cohort of 891 AIS patients and found that nearly half (48.5%) complained of back pain. This rate is higher than most previously published reports on AIS as well as reported rates of back pain in adolescents without scoliosis. Pain was most common in the lumbar region, especially among patients with lumbar curves. Lumbar pain also tended to be more severe than pain in other regions. The presence and severity of pain were not associated with curve magnitude, progression to surgery, Lenke curve type, Vitamin D deficiency, or sex. Pain was more common among older and more skeletally mature patients as well as those with government insurance. At our center, patients with back pain were frequently referred to physical therapy, though further research is needed to determine the benefit of therapy and to compare various therapy methods. We recommend that surgeons view back pain as a common part of AIS and counsel patients that they have higher risk of back pain than the general population, especially those groups identified above. We also encourage further research into the socioeconomic factors leading to higher rates of back pain among Medicaid patients.

## Data Availability

The data that support the findings of this study are not openly available due to reasons of sensitivity and are available from the corresponding author upon reasonable request. Data are located in controlled access data storage via password protected REDcap database at LSUHSC.
